# Retrospective Comparison of Intermediate-term Efficacy of 350 mm^2^ Glaucoma Drainage Implants and Medium-sized 230-250 mm^2^ Implants

**DOI:** 10.5005/jp-journals-10008-1214

**Published:** 2017-01-18

**Authors:** Alissa M Meyer, Cooper D Rodgers, Baiming Zou, Nicole C Rosenberg, Aaron D Webel, Mark B Sherwood

**Affiliations:** 1 Research Assistant, Department of Ophthalmology, University of Florida Gainesville, Florida, USA; 2 Research Assistant, Department of Ophthalmology, University of Florida Gainesville, Florida, USA; 3 Research Assistant and Professor, Department of Biostatistics, University of Florida, Gainesville Florida, USA; 4 Research Assistant, Department of Ophthalmology, University of Florida Gainesville, Florida, USA; 5 Resident, Department of Ophthalmology, University of Florida Gainesville, Florida, USA; 6 Professor, Department of Ophthalmology, University of Florida Gainesville, Florida, USA

**Keywords:** Baerveldt, Glaucoma, Glaucoma drainage device, Intraocular pressure, Molteno, Retrospective study, Visual acuity.

## Abstract

**Aim:**

To compare the intermediate-term efficacy of a large surface area Baerveldt 350 mm^2^ glaucoma drainage device (GDD) with medium surface area implants (Baerveldt 250 mm^2^ and Molteno 3, 230, or 245 mm^2^).

**Design:**

This is a retrospective, nonrandomized comparative trial.

**Materials and methods:**

A total of 94 eyes of 94 patients of mixed glaucoma diagnoses without any prior glaucoma surgical procedures and who had undergone a glaucoma drainage implant surgery with either a large Baerveldt 350 mm^2^ GDD or a medium-sized GDD (Baerveldt 250 mm^2^ or Molteno 230 or 245 mm^2^) were reviewed for intraocular pressure (IOP), number of glaucoma medications, and visual acuity (VA) preoperatively, and at 1, 2, and 3 years postprocedure.

**Results:**

No significant differences were found in mean IOP, number of glaucoma medications used, and VA at 1, 2, and 3 years postoperatively. The rate of additional glaucoma procedures was similar between the two groups.

**Conclusion:**

There is no clear evidence that a larger implant surface area beyond 230 to 250 mm^2^ is advantageous in providing intermediate-term IOP control.

**Clinical significance:**

It may be technically easier to surgically place a GDD that does not need to have its wings placed underneath the recti muscles, and the IOP results are similar.

**How to cite this article:**

Meyer AM, Rodgers CD, Zou B, Rosenberg NC, Webel AD, Sherwood MB. Retrospective Comparison of Intermediate-term Efficacy of 350 mm^2^ Glaucoma Drainage Implants and Medium-sized 230-250 mm^2^ Implants. J Curr Glaucoma Pract 2017;11(1):8-15.

## INTRODUCTION

For many decades, the primary surgery for lowering intraocular pressure (IOP) in patients with glaucoma has been trabeculectomy. If this fails, it is possible to either revise the original trabeculectomy site or create a new site, but there is only a limited amount of area close to the limbus for additional procedures. Inferior trabecu-lectomies were shown to have a significantly increased risk of blebitis and endopthalmitis^1^ and are now rarely performed. Repeat trabeculectomies generally have a poorer success rate than initial trabeculectomies due to increased risk of scarring of the bleb.^2-4^

Drainage implant surgery delivers aqueous humor from the anterior chamber via a small silicone tube to a nonscarred, more posterior location between the muscles. The procedure was originally recommended for patients at a high risk of scarring trabeculectomy blebs, including neovascular glaucoma, uveitic and other secondary glaucomas, and pseudophakic/aphakic glaucomas. Early implants, like the Schocket and Molteno double-plate implant, often utilized more than one quadrant for creating a bleb over an episcleral plate, and a prospective randomized study by Heuer et al^5^ showed better IOP control at 1 and 2 years with a double-plate Molteno than a single-plate Molteno. This led to the concept that a larger surface area may be important in achieving better IOP control with drainage implants. Nowadays, all modern implants utilize just a single quadrant for the episcleral plate. There are different-sized implants, but it is unclear whether the assumption that bigger implants provide better IOP long-term is correct.

The prospective randomized Ahmed Baerveldt Comparison (ABC) study and the prospective randomized Ahmed *vs* Baerveldt (AVB) study both showed better efficacy for controlling IOP with the Baerveldt 350 mm^2^ implant than with the Ahmed FP7 (184 mm implant).^6,7^ Although this difference may be related to the larger surface area of the Baerveldt implant, it should also be noted that the Ahmed implant is valved and delivers aqueous to the episcleral plate area immediately after surgery, whereas the Baerveldt is a nonvalved implant and is generally occluded until a capsule forms over the plate. This difference in the timing of aqueous delivery has been suggested by some as a possible factor influencing the thickness of the capsule formed over the plate due to the increased cytokines in the aqueous in the early postoperative period.^8,9^

Some retrospective studies comparing different-sized nonvalved implants have indicated that implant plate size may not be a significant factor in the success of glaucoma drainage implant surgery. An early retrospective study by Smith et al^10^ comparing double-plate Molteno implants (270 mm^2^ surface area) with Baerveldt 350 mm^2^ implants showed no significant differences in IOP control or success rate. A larger, recent comparative case study done by Allan et al^11^ found no difference in surgical failure rate or the visual acuity (VA), mean IOP, and the number of glaucoma medications being taken at the final follow-up visit between Baerveldt implants of 250 and 350 mm^2^ surface areas. An earlier comparative trial performed by Seah et al^12^ also found no significant difference between the Baerveldt 250 and 350 mm^2^ in success rate (maintaining an IOP between 6 and 21 mm Hg), complication rate, IOP, VA, and the number of medications at the final visit. These last two retrospective studies had a mean follow-up period of 31 to 40 months and included a range of diagnoses, including subjects with high-risk disease or previous trabeculectomy failure.

We have undertaken a retrospective study to further compare outcomes between patients receiving a large surface area glaucoma drainage implant (350 mm^2^) *vs* those receiving a medium-sized implant of approximately 230 to 250 mm^2^ surface area.

## MATERIALS AND METHODS

All research performed in this retrospective study was in accordance with the University of Florida’s Institutional Review Board (IRB) with protocol approval prior to initiation of the study. With the IRB’s permission, a waiver of informed consent was not obtained for the collection or use of these data. This study adhered to the tenets of the Declaration of Helsinki and all federal and state laws.

This study was a retrospective chart review of glaucoma patients who underwent glaucoma drainage device (GDD) implantation surgery by a single surgeon at the University of Florida, Gainesville, Florida, between February 18, 1999, and April 20, 2015. Initially, 350 patients were identified from hospital records, and we were able to locate 277 of these records as hardcopy files at the University of Florida Health Eye Center. The study was then refined to include only patients who received either a Baerveldt 350 mm^2^ or Baerveldt 250 mm^2^ implant (Abbott Laboratories, Inc. Abbott Park, IL, USA) or a Molteno 245 mm^2^ or Molteno 230 mm^2^ implant (Molteno® Ophthalmic Ltd., Dunedin, NZ) and who had no prior failed trabeculectomy or GDD surgery. Ten patients had two eyes that qualified for inclusion, and in those cases, only the first eye that received an implant was included. The final study list included 94 eyes of 94 patients.

For these eyes, demographic information at the time of surgery was recorded, including gender, race, age at time of surgery, lens status (pseudophakic, phakic, or aphakic), study eye (right or left), the study eye’s glaucoma diagnosis, and the implant used. The Baerveldt 350 mm^2^ implant comprised the large plate-size group, and the Baerveldt 250 mm^2^, Molteno 245 mm^2^, and Molteno 230 mm^2^ implants made up the medium plate size group. Glaucoma diagnoses were grouped into two categories: Primary glaucomas [primary open angle glaucoma, pseu-doexfoliation, and pigmentary glaucoma] and secondary glaucomas (caused by uveitis, angle closure, neovascu-larization, or other conditions).

The IOP, VA, and the number of glaucoma medication groups being taken were recorded from the clinic visits that were presurgical and at 1, 2, and 3 years postopera-tion. The glaucoma care visit closest to 2 weeks before surgery, without going further past, was used to obtain presurgical data. If no visit between 2 weeks and the day of surgery was found, as was the case for eight of the subjects, the next most recent pre-op glaucoma care visit was used. Additional postoperative glaucoma procedures that marked the implant as a failure, which were diode cyclophotocoagulation (CPC) treatments or a second GDD implant (n), ended data collection for that eye.

### Statistical Analysis

To compare the demographic characteristics between the large and medium plate size groups, we conducted two sample t-tests and χ^2^ tests by using the GraphPad statistical software package (GraphPad Software, Inc., La Jolla, CA, USA). When comparing ethnicity between the large and medium plate size groups, the African American and Hispanic/Asian categories were combined. To investigate the average IOP, number of medications, and VA difference at years 1, 2, and 3 between the large and medium plate size groups, we performed univariate analysis by conducting two-sample t-tests for continuous outcomes, such as IOP, and χ^2^ tests for categorical data, such as ranges of IOP and VA, etc.

Snellen scale VA measurements that were between the lines on the Snellen chart (+/- letters on a line) were rounded to the last complete line the patient could read. VA measurements were converted from Snellen to logarithm of the minimum angle of resolution (LogMAR) values to better measure the average VA.^13^ Patients who were measured as “count fingers” were converted using the table published by Wendy Strouse Watt.^14^ Patients who were measured as “hand motion” were assigned a LogMAR value of 4.

The difference between medium and large plate size implants for IOP, VA, and Med number was further evaluated by fitting multiple regression models to adjust for the confounding factors including age, gender, type of glaucoma, operated eye (left or right), pseudophakia status, and baseline measurements using the open source R statistical software (Free Software Foundation, Boston, MA, USA).

### RESULTS

A Baerveldt 350 mm^2^ drainage implant was placed in 52 eyes, a Baerveldt 250 mm^2^ in 16 eyes, and 26 eyes received a Molteno 230 or 245 mm^2^. Based on the demographic data collected, there were no significant differences in age, gender, ethnicity, glaucoma diagnosis, study eye, and lens status between the patients who had received a medium plate size implant or a large plate size implant (Table 1). The ratio of primary glaucomas to secondary glaucomas was not significantly different between the groups. However, all 11 neovascular glaucoma eyes were in the medium plate size group (Table 2).

Nine study eyes ended in surgical failure before 3 years after their drainage implant surgery - one had a second GDD implantation and the other eight underwent diode laser CPC. Four of the CPC recipients and the second GDD recipient were in the large-sized GDD group, while the other four CPC surgical failures were in the medium group (p = 1.0) (Table 1).

**Table 1:** **Table 1:** Demographics for the large plate size group and medium plate size group

*Variable*		*Subgroup*		*Large GDD plate size group (n = 52)*		*Medium GDD plate size group (n = 42)*		*p-value*	
GDD implant (n)		Baerveldt 350 mm^2^		52				N/A	
		Baerveldt 250 mm^2^				16			
		Molteno 245 mm^2^				11			
		Molteno 230 mm^2^				15			
Gender		Male		29		25		0.71^a^	
		Female		23		17			
Ethnicity		Caucasian		39		34		0.49^a^	
		African American		13		5			
		Hispanic/Asian		0		3			
Glaucoma diagnosis		Primary glaucomas		27		18		0.38^a^	
		Secondary glaucomas		25		24			
Study eye		Right		27		22		0.96^a^	
		Left		25		20			
Lens status		Pseudophakic		36		22		0.18^a^	
		Phakic		10		15			
		Aphakic		6		5			
Average age (SD)				66.5 (16.6)		62.4 (15.9)		0.23^b^	
Number of failures requiring surgical intervention (%)				5 (9.6%)		4 (9.5%)		1.0^b^	

^a^p-value was found with a χ^2^ test; ^b^p value was found with a two-sample t-test; SD: Standard deviation

**Table 2:** **Table 2:** Glaucoma diagnoses of the large plate size group and medium plate size group

*Glaucoma category*		*Specific glaucoma* *diagnosis*		*Large GDD plate size* *group (n = 52)*		*Medium GDD plate size* *group (n = 52)*		*total*	
Primary		Primary open angle		23		14		37	
		Low tension		1				1	
		Ocular hypertension				1		1	
		Pigmentary				1		1	
		Pseudoexfoliative		3		2		5	
		Total		27		18		45	
Secondary		Angle closure		6		4		10	
		Angle recession/trauma		5		2		7	
		Secondary open angle		2		1		3	
		Uveitic		9		5		14	
		Congenital		3		1		4	
		Neovascular				11		11	
		Total		25		24		38	

**Graph 1:**
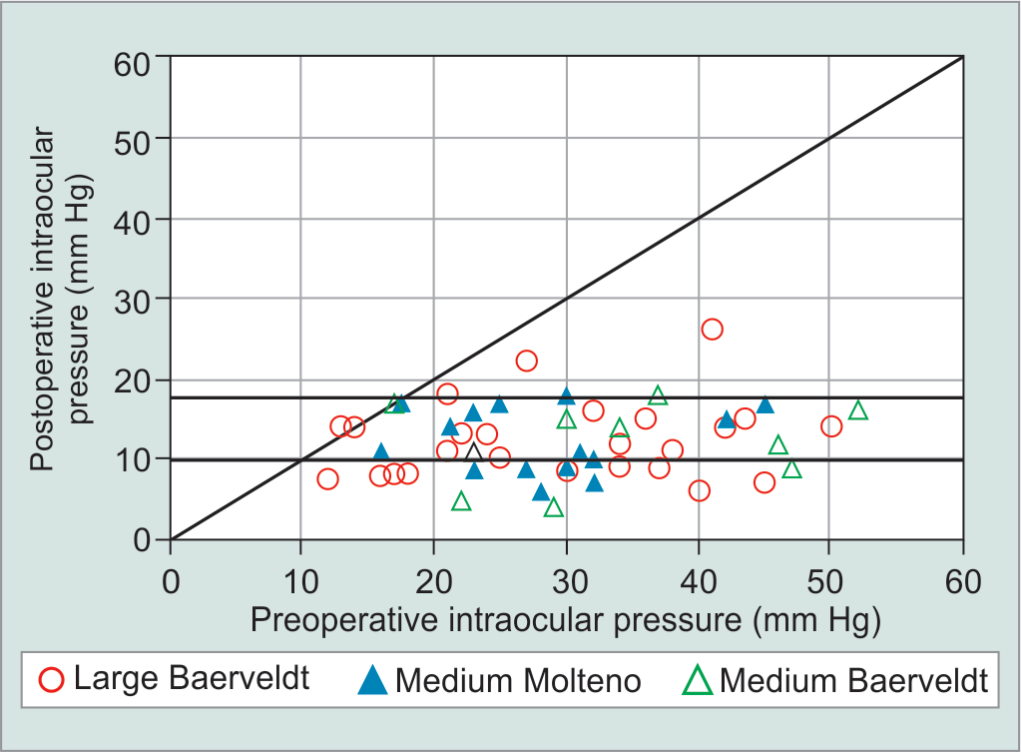
Scatterplot showing the similarity in distribution of IOP at 2 years of follow-up *vs* that at baseline for the large Baerveldt 350 mm^2^ implant, medium Baerveldt 250 mm^2^ implant, and medium Molteno 230 and 245 mm^2^ implant

The mean preoperative IOP was slightly higher in the medium plate size group, although not statistically significantly different (p = 0.53) and was similarly higher at years 1 and 3, but again not significantly different (Table 3A). At 2 years postoperatively, the pressures were almost identical at 12.4 and 12.5 mm Hg in the large and medium plate groups respectively (p = 0.92). After adjusting for potential confounding factors of age, gender, type of glaucoma, eye (left or right), lens status, and baseline IOP, the differences were still nonsignificant (p > 0.2). A scatterplot showing the difference in IOP at 2 years postoperative compared with preopera-tive levels shows little separation of the groups, with all but three eyes achieving pressures of 18 mm Hg or less (Graph 1).

Preoperatively, the mean number of medications used was slightly higher in the large plate group than the medium plate group, although it was not statistically different (p = 0.31), and was slightly lower at all 3 years of follow-up, with a trend toward significance at year 2 (Table 3B). After adjusting for potential confounding factors, there was a significant difference at year 3 (p = 0.024) in favor of the large plate group, but only a borderline trend at years 1 and 2.

The mean VA was slightly better preoperatively in the large plate group than the medium plate group, but again not statistically significantly different (p = 0.33) (Table 3C). Postoperatively, there was a similar difference in mean LogMAR between the groups, which was not statistically different, even when adjusting for potential confounding factors. When the number of eyes that showed two or more Snellen lines of decreased VA was compared, there was no difference between the groups (Table 4). Of the eyes with two or more Snellen lines improved vision in the large plate group, two had cataract surgery in the postoperative period (one combined with Descemet stripping automated endothelial keratoplasty) and two eyes had a corneal surgery and one had a yttrium aluminum garnet capsulotomy. In the medium plate group, one patient had an intraocular lens exchange and two had corneal surgery.

**Table 3A:** **Table 3A:** Effect of implant plate size on IOP

		*Mean IOP (mm Hg)*									
*Time*		*Large plate (n)*		*Medium plate (n)*		*Difference (mm Hg)*		*Std. error of diff.*		*p-value^a,b^*	
Pre-op		30.0 (52)		31.4 (42)		–1.4		*2.2*		0.53	
Post-op 1 year		12.6 (38)		13.7 (37)		–1.1		1.1		0.33	
Post-op 2 years		12.4 (25)		12.5 (27)		–0.1		1.3		0.92	
Post-op 3 years		10.7 (17)		12.3 (21)		–1.6		1.5		0.27	

^a^p-value was found with a two-sample t-test; ^b^After adjusting for the confounding factors of age, gender, type of glaucoma, eye (left or right), lens status, and baseline IOP, the differences were still nonsignificant (p > 0.2)

**Table 3B:** **Table 3B:** Effect of implant plate size on mean number of glaucoma meds

		*Mean # of medications*									
*Time*		*Large plate (n)*		*Medium plate (n)*		*Difference (# meds)*		*Std. error of diff.*		*p-value^a,b^*	
Pre-op		3.0 (52)		2.8 (42)		0.2		0.2		0.31	
Post-op 1 year		1.7 (39)		2.0 (37)		–0.3		0.2		0.20	
Post-op 2 years		1.6 (26)		2.1 (27)		–0.5		0.3		0.07	
Post-op 3 years		1.4 (18)		2.0 (21)		–0.6		0.3		0.10	

^a^p-value was found with a two-sample t-test; ^b^After adjusting for the confounding factors of age, gender, type of glaucoma, eye (left or right), lens status, and baseline meds, the differences were significant only at year 3 (p = 0.024), but of borderline trend at years 1 and 2 (p = 0.10 and p = 0.09 respectively)

**Table 3C:** **Table 3C:** Effect of implant plate size on LogMAR VA

		*Mean VA (LogMAR units)*									
*Time*		*Large plate (n)*		*Medium plate (n)*		*Difference (LogMAR)*		*Std. error of diff.*		*p-value^a,b^*	
Pre-op		1.3 (51)		1.5 (42)		–0.2		0.2		0.33	
Post-op 1 year		1.0 (38)		1.2 (37)		–0.2		0.2		0.30	
Post-op 2 years		1.0 (25)		1.4 (27)		–0.4		0.3		0.24	
Post-op 3 years		1.03 (17)		1.26 (21)		–0.2		0.4		0.52	

^a^p-value was found with a two-sample t-test; ^b^After adjusting for the confounding factors of age, gender, type of glaucoma, eye (left or right), lens status, and baseline VA, there was a borderline trend to better vision with the medium plates at 1 year (p = 0.07), but no significance at years 2 and 3 (p = 0.5-0.7)

For eyes with advanced glaucoma, which may require a goal of very low normal or subnormal IOPs (10 mm Hg or less), there was no significant difference in achieving this goal between the large and medium plate size groups (Table 5).

In case the material of the implant might have an effect on postoperative pressure control, a subanalysis was performed directly comparing the Baerveldt 350 and 250 mm^2^ implants, which have the same plate material and manufacturing processes. There were no significant differences in IOP or number of meds in any of the 3 years of follow-up between these different-sized implants (p > 0.2) (Appendix 1). The VA of the Baerveldt 350 mm^2^ implant was significantly better than the 250 mm^2^ implant preoperatively and at every year of follow-up (Appendix 1). After adjusting for potential confounding factors when comparing the two different-sized Baerveldt implants, the differences were nonsignificant for VA (p > 0.1).

Patients with neovascular glaucoma comprised 26% of the medium plate group, but there were none in the large-sized group. Given the difference in the distribution of these patients, the analyses in Tables 3A to C were repeated with neovascular patients excluded, since historically patients with this diagnosis might have a poorer outcome and this might be a confounding factor. After exclusion of the neovascular glaucoma patients, no differences were found from the original analysis and there were still no significant differences between the large- and medium-sized implant groups for IOP control, medication use, or VA at 1, 2, and 3 years postoperative (Appendix 2).

## DISCUSSION

Two recent prospective, multicenter, randomized studies (ABC and AVB) compared Baerveldt 350 mm^2^ drainage implants with the Ahmed FP7 184 mm^2^ implant and found a statistically significant difference in the efficacy of the implants to achieve low postoperative IOP.^6,7^ The 5-year results of the ABC study showed a mean IOP in the Ahmed group of 14.7 (± 4.4) mm Hg and 12.7 (± 4.5) mm Hg in the Baerveldt group (p = 0.015).^6^ The AVB study’s 5-year results showed a mean IOP of 16.6 (± 5.9) mm Hg for the Ahmed and 13.6 (± 5.0) mm Hg for the Baerveldt (p = 0.001) groups.^7^ As well as having a difference in implant size, there is also a difference in timing of aqueous reaching the episcleral plate area because the Ahmed implant is valved and delivers aqueous immediately to the plate, whereas the Baerveldt is generally occluded for several weeks postoperatively to prevent overdrainage.

In our study, we found IOP results for the 350 mm^2^ Baerveldt implant of 12.6, 12.4, and 10.7 mm Hg at 1, 2, and 3 years respectively, which were similar to the above randomized studies. The paper by Seah et al,^12^ which retrospectively compared the 250 mm^2^ with the 350 mm^2^ Baerveldt implants in Asian eyes, and the paper by Allan et al,^11^ which compared these implants in the United States, were similar to our study results and found no significant difference in mean IOP levels between the 350 mm^2^ and the smaller implants. Both these studies and our study contained a mixed group of diagnoses, including primary glaucomas, secondary glaucomas, and neovascular glaucoma.^11,12^ After adjusting for gender, preoperative IOP, and length of follow-up, Seah et al^12^ found that as well as increasing age, the number of previous operations performed before implant surgery positively correlated with failure. In our study, patients with prior glaucoma surgeries (trabeculectomies and glaucoma drainage implant surgeries) were excluded.

**Table 4:** **Table 4:** Univariate analysis for tube size effect on VA

		*Large GDD plate size, % (n)*						*Medium GDD plate size, % (n)*							
*Years post-op*		*Decreased ^b^*		*Improved ^c^*		*Stable^d^*		*Decreased ^b^*		*Improved^c^*		*Stable^d^*		*p-value^a^*	
1		13% (5)		23% (9)		64% (25)		13% (5)		19% (7)		68% (25)		0.49	
2		15% (4)		31% (8)		54% (14)		15% (4)		11% (3)		74% (20)		0.19	
3		17% (3)		39% (7)		44% (8)		23% (5)		18% (4)		59% (13)		0.34	

^a^p-value was found with a χ^2^ test; ^b^Decreased: Percentage of eyes that decreased 2 or more Snellen lines from preoperative VA; ^c^Improved: Percentage of eyes that gained 2 or more Snellen lines from preoperative VA; ^d^Stable: Percentage of eyes within 1 Snellen line of preoperative VA

**Table 5:** **Table 5:** Univariate analysis for tube size effect on IOP: The percentage of eyes achieving an IOP of 10 mm Hg or less, between 11 and 21, and 22 mm Hg or greater for large and medium-sized implant plate types

		*Large GDD plate size, % (n)*						*Medium GDD plate size, % (n)*							
*Time*		*IOP ≤ 10*		*10 < IOP < 22*		*22 ≤ IOP*		*IOP ≤ 10*		*10 < IOP < 22*		*22 ≤ IOP*		*p-value^a^*	
Pre-op		0		27% (14)		73% (38)		0		17% (7)		83% (35)		0.24	
Post-op 1 year		37% (14)		58% (22)		5% (2)		22% (8)		76% (28)		3% (1)		0.26	
Post-op 2 years		40% (10)		52% (13)		8% (2)		37% (10)		63% (17)		0		0.83	
Post-op 3 years		53% (9)		47% (8)		0		33% (7)		67% (14)		0		0.22	

^a^p-value was found with a χ^2^ test

The AVB study noted significantly less medication use in the Baerveldt 350 mm^2^ than the Ahmed at 5 years (p = 0.033), but in the ABC study there was no significant difference.^6,7^ In the paper by Seah et al,^12^ Allan et al,^11^ and our study, no significant difference was found in medication use at 1 year. No significant differences in mean VA were noted between groups in either of the prospective randomized studies comparing Baerveldt implants to Ahmed implants or in the retrospective studies by Seah, Allan, or our investigation.^6,7,9,10^

Limitations for our study include the fact that it is retrospective and, thus, is subject to bias from lack of randomization. However, demographically, the two groups were not statistically different, except for the allocation of neovascular glaucoma patients to the medium-sized group. As noted, when these were excluded, there were still no differences in IOP, medication use, or VA between the larger and medium-sized implants. Other limitations include loss to follow-up and relatively small sample size, which will affect the power of the study to detect differences.

## CONCLUSION

In conclusion, we did not find any significant differences between the larger 350 mm^2^ Baerveldt implants where the wings of the implant are placed beneath the recti muscles compared with the smaller 230 to 250 mm^2^ implants, suggesting that an optimal size may have been achieved even with these smaller surface area implants that are technically easier to insert. Previous literature suggests that even smaller implants like the single-plate Molteno (135 mm^2^) and very large implants, such as the Baerveldt 500 mm^2^ may have poorer IOP outcomes than those in this middle surface area range.^5,15^

## CLINICAL SIGNIFICANCE

It may be more technically demanding to surgically place a GDD with wings that need to be inserted underneath the recti muscles, such as with the Baerveldt 350 mm^2^ implant. Based on the results of this study, the postoperative IOP, medication, and VA results are likely to be similar to the smaller class of GDDs including the Baerveldt 250 mm^2^ and the 230 to 245 mm^2^ Molteno 3.

## APPENDIX 1

**Table:** Effect of Baerveldt 250 *vs* 350 mm^2^ on IOP

		*Mean IOP (mm Hg)*									
*Time*		*Baerveldt 350 mm^2^ (n)*		*Baerveldt 250 mm^2^ (n)*		*Difference (mm Hg)*		*Std. error of diff.*		*p-value^a^*	
Pre-op		30.0 (52)		32.7 (16)		–2.7		3.3		0.42	
Year 1 post-op		12.6 (38)		13.7 (15)		–1.1		1.4		0.44	
Year 2 post-op		12.4 (25)		12.6 (12)		–0.2		1.7		0.90	
Year 3 post-op		10.7 (17)		12.6 (5)		–2.0		2.2		0.38	

^a^p-value was found with a two-sample t-test

**Table:** Effect of Baerveldt 250 *vs* 350 mm^2^ on the mean number of glaucoma meds

		*Mean # medications*									
*Time*		*Baerveldt 350 mm^2^ (n)*		*Baerveldt 250 mm^2^ (n)*		*Difference (# meds)*		*Std. error of diff.*		*p-value^a^*	
Pre-op		3.0 (52)		2.6 (15)		0.4		0.3		0.28	
Year 1 post-op		1.7 (39)		1.5 (16)		0.1		0.3		0.67	
Year 2 post-op		1.6 (26)		1.8 (12)		–0.2		0.3		0.54	
Year 3 post-op		1.4 (18)		1.2 (5)		0.2		0.5		0.73	

^a^p-value was found with a two-sample t-test

**Table:** Effect of Baerveldt 250 *vs* 350 mm^2^ on LogMAR VA

		*Mean VA (LogMAR units)*									
*Time*		*Baerveldt 350 mm^2^ (n)*		*Baerveldt 250 mm^2^ (n)*		*Difference (LogMAR)*		*Std. error of diff.*		*p-value^a^*	
Pre-op		1.3 (51)		1.9 (15)		–0.7		0.3		0.043	
Year 1 post-op		1.0 (38)		1.7 (16)		–0.7		0.3		0.019	
Year 2 post-op		1.0 (25)		2.1 (12)		–1.1		0.4		0.0057	
Year 3 post-op		1.03 (17)		2.3 (5)		–1.2		0.5		0.034	

^a^p-value was found with a two-sample t-test

## APPENDIX 2

**Table:** Effect of implant plate size on IOP, with neovascular subjects omitted

		*Mean IOP (mm Hg)*									
*Time*		*Large plate (n)*		*Medium plate (n)*		*Difference (mm Hg)*		*Std. error of diff.*		*p-value^a^*	
Pre-op		30.0 (52)		30.1 (31)		–0.1		2.4		0.97	
Year 1 post-op		12.6 (38)		14.1 (27)		–1.4		1.2		0.23	
Year 2 post-op		12.4 (25)		11.7 (19)		0.6		1.4		0.65	
Year 3 post-op		10.7 (17)		12.1 (17)		–1.5		1.5		0.34	

^a^p-value was found with a two-sample t-test

**Table:** Effect of implant plate size on the mean number of glaucoma meds, with neovascular subjects omitted

		*Mean # of medications*									
*Time*		*Large plate (n)*		*Medium plate (n)*		*Difference (# meds)*		*Std. error of diff.*		*p-value^a^*	
Pre-op		3.0 (52)		2.7 (31)		0.3		0.2		0.30	
Year 1 post-op		1.7 (39)		2.0 (27)		–0.4		0.2		0.12	
Year 2 post-op		1.6 (26)		2.1 (19)		–0.5		0.3		0.11	
Year 3 post-op		1.4 (18)		2.1 (17)		–0.7		0.3		0.057	

^a^p-value was found with a two-sample t-test

**Table:** Effect of implant plate size on LogMAR VA, with neovascular subjects omitted

		*Mean VA (LogMAR units)*									
*Time*		*Large plate (n)*		*Medium plate (n)*		*Difference (LogMAR)*		*Std. error of diff.*		*p-value^a^*	
Pre-op		1.3 (51)		1.1 (31)		0.2		0.2		0.44	
Year 1 post-op		1.0 (38)		0.8 (27)		0.1		0.2		0.52	
Year 2 post-op		1.0 (25)		0.9 (19)		0.1		0.3		0.71	
Year 3 post-op		1.03 (17)		1.0 (17)		0.2		0.3		0.94	

^a^p-value was found with a two-sample t-test
